# Pre-Exposure of Foodborne Staphylococcus aureus Isolates to Organic Acids Induces Cross-Adaptation to Mild Heat

**DOI:** 10.1128/spectrum.03832-22

**Published:** 2023-03-14

**Authors:** Xinyu Liao, Xin Chen, Anderson S. Sant'Ana, Jinsong Feng, Tian Ding

**Affiliations:** a Department of Food Science and Nutrition, Zhejiang University, Hangzhou, Zhejiang, China; b School of Mechanical and Energy Engineering, NingboTech University, Ningbo, China; c Future Food Laboratory, Innovation Center of Yangtze River Delta, Zhejiang University, Jiashan, China; d Department of Food Science and Nutrition, Faculty of Food Engineering, University of Campinas, Campinas, SP, Brazil; Institut National de Santé Publique du Québec

**Keywords:** acid-heat cross-adaptation, cell membrane, food safety, *Staphylococcus aureus*, stress response

## Abstract

Staphylococcus aureus is a typical enterotoxin-producing bacterium that causes food poisoning. In the food industry, pasteurization is the most widely used technique for food decontamination. However, pre-exposure to an acidic environment might make bacteria more resistant to heat treatment, which could compromise the bactericidal effect of heat treatment and endanger food safety. In this work, the organic acid-induced cross-adaptation of S. aureus isolates to heat and the associated mechanisms were investigated. Cross-adaptation area analysis indicated that pre-exposure to organic acids induced cross-adaptation of S. aureus to heat in a strain-dependent manner. Compared with other strains, S. aureus strain J15 showed extremely high heat resistance after being stressed by acetic acid, citric acid, and lactic acid. S. aureus strains J19, J9, and J17 were found to be unable to develop cross-adaptation to heat with pre-exposure to acetic acid, citric acid, and lactic acid, respectively. Analysis of the phenotypic characteristics of the cell membrane demonstrated that the acid-heat-cross-adapted strain J15 retained cell membrane integrity and functions through enhanced Na^+^K^+^-ATPase and F_o_F_1_-ATPase activities. Cell membrane fatty acid analysis revealed that the ratio of anteiso to iso branched-chain fatty acids in the acid-heat-cross-adapted strain J15 decreased and the content of straight-chain fatty acids exhibited a 2.9 to 4.4% increase, contributing to the reduction in membrane fluidity. At the molecular level, *fabH* was overexpressed with preconditioning by organic acid, and its expression was further enhanced with subsequent heat exposure. Organic acids activated the GroESL system, which participated in the heat shock response of S. aureus to the subsequent heat stress.

**IMPORTANCE** Cross-adaptation is one of the most important phenotypes in foodborne pathogens and poses a potential risk to food safety and human health. In this work, we found that pretreatment with acetic acid, citric acid, and lactic acid could induce subsequent heat tolerance development in S. aureus. Various S. aureus strains exhibited different acid-heat cross-adaptation areas. The acid-induced cross-adaptation to heat might be attributable to membrane integrity maintenance, stabilization of the charge equilibrium to achieve a normal internal pH, and membrane fluidity reduction achieved by decreasing the ratios of anteiso to iso fatty acids. The *fabH* gene, which is involved in fatty acid biosynthesis, and groES/groEL, which are related to heat shock response, contributed to the development of the acid-heat cross-adaptation phenomenon in S. aureus. The investigations of the stress cross-adaptation phenomenon in foodborne pathogens could help optimize food processing to better control S. aureus.

## INTRODUCTION

Staphylococcus aureus is a common Gram-positive bacterium. It can survive in a pH range of 4.2 to 9.3 and a temperature range of 7°C to 48.5°C (1). Meat, poultry, eggs, milk and dairy products are the primary food sources of S. aureus (2). The ingestion of food contaminated by staphylococcal enterotoxins results in food poisoning, with symptoms such as nausea, stomach cramps, diarrhea, and vomiting (3). Food contamination caused by S. aureus is a significant concern in public health worldwide (4). According to the European Food Safety Authority (EFSA), staphylococcal enterotoxins were listed as one of the top four bacterial toxins causing foodborne outbreaks (27.8% of the total) in the European Union in 2020 (5). In the United States, it is estimated that S. aureus toxins were responsible for 1,019 illnesses and 127 hospitalizations from 1998 to 2015 and accounted for 12.8% of all foodborne outbreaks attributed to pork (6).

Acidification is one of the commonly used strategies to control the growth of pathogenic bacteria and inhibit food spoilage (7). Foods such as fruits and vegetables naturally contain citric acid, malic acid, and other organic acids. Organic acids can stabilize the pH between 3.5 and 4.9, making the food matrix weakly acidic (7, 8). In the process of food production, acidic ingredients are widely used as food additives to improve the flavor of products and as preservatives to extend the shelf life of food. The widely used preservatives in the food industry include lactic acid, citric acid, sorbic acid, and benzoic acid (9). In the process of slaughtering, organic acids are usually sprayed on the surface of carcasses to prevent microbial contamination (10, 11). With the occurrence of glycolysis in the meat matrix, pyruvate, lactic acid, and other acidic substances are produced, forming a weakly acidic environment (12).

Under external stress, pathogenic bacteria can initiate a defensive response and develop stress resistance (13, 14). A growing number of studies have found that in addition to a single stress response, initial exposure to one specific stress can confer resistance to subsequent similar or heterogeneous stress to bacteria (15, 16). Under sublethal stress, bacteria activate the stress response to help repair damage and maintain cellular homeostasis, thus enhancing the tolerance of the bacteria to other adversities, known as cross-adaptation (15, 17). It has been reported that traditional food processing and storage methods can induce cross-adaptation of S. aureus. Cebrián et al. (18) found that the combinations acid-heat, acid-H_2_O_2_, alkali-H_2_O_2_, heat-acid, and heat-H_2_O_2_ initiated the cross-adaptation phenomenon in S. aureus. In another study by Cebrián et al. (19), acid, alkali, H_2_O_2_, NaCl, heat, and cold stresses were used to pretreat S. aureus for a short period (2 h), and the results showed that combined treatment with high temperature and alkali stress could induce S. aureus to resist subsequent exposure to a pulsed electric field. Cross-adaptation may enable bacteria to endure food processing, potentially endangering food safety. To date, the mechanisms of cross-adaptation are still unclear, and several hypotheses have been proposed. The bacterial cell membrane serves as the first line of defense against external stress, and numerous physiological activities of bacteria are carried out on the cell membrane (20). Fatty acids are a crucial part of cell membranes because they ensure the integrity, permeability, and fluidity of the membrane and are directly linked to the bacterial stress response. Fatty acids play vital roles in maintaining the stability of the intracellular environment. In addition to the regulation of bacterial cell membranes, foodborne pathogenic bacteria have the ability to sense the external stress environment and reprogram cells to change the internal environmental conditions (21).

In this study, the effect of acid pretreatments (acetic acid, citric acid, l-lactic acid) on the thermal resistance of various S. aureus isolates from foods was quantitatively analyzed. The strains that developed cross-adaptation were identified by screening, and the mechanisms underlying the acid-heat cross-adaptation were further explored at the cellular and molecular levels.

## RESULTS AND DISCUSSION

### Cross-adaptation of acid-pretreated S. aureus to heat.

The cross-adaptation area was first proposed by Lou and Yousef (22) in 1997 and applied to compare the survival capacity of strains in the cross-adapted state. The acid-heat cross-adaptation area (expressed in minutes·log_10_ CFU) equals the area under acid-heat treatment curve minus the area under heat stress alone treatment curve. A positive value of cross-adaptation area indicates that preadaptation to acid stress enhances the survival of S. aureus under heat stress. As shown in Table 1, the magnitude of the acid-heat cross-adaptation area was affected by the types of organic acids and the strains of S. aureus. Under the same organic acid stress, various S. aureus strains showed different areas of cross-adaptation to heat stress. After being subjected to acetic acid, citric acid, and lactic acid, S. aureus strain J15 exhibited strong resistance to the subsequent heat stress with cross-adaptation regions of 6.910, 6.870, and 6.360 min·log_10_ CFU, respectively, significantly higher than those of other S. aureus strains. An acid-heat cross-adaptation area with a negative value indicates that pre-exposure to organic acid makes S. aureus more susceptible to heat. The minimum cross-adaptation areas were −0.584, −1.525, and −0.107 min·log_10_ CFU for S. aureus strains J19, J9, and J17 upon the acetic acid, citric acid, and lactic acid pretreatments, respectively (Table 1). Based on the values of the cross-adaptation region, S. aureus strain J15 was selected as the strain capable of undergoing acid-heat cross-adaptation (acid-heat-cross-adapted strain), while S. aureus strains J19, J9, and J17 were selected as control strains not exhibiting acetic acid-, citric acid-, and lactic acid-heat cross-adaptation.

**TABLE 1:** Comparisons of areas of cross-adaptation of various Staphylococcus aureus strains to organic acid-heat stress

Strain	Cross-adaptation region size (min·log_10_ CFU^*a*^)		
	Acetic acid-heat	Citric acid-heat	Lactic acid-heat
J1	3.694 ± 0.114 B,c	5.166 ± 0.127 A,b	2.772 ± 0.127 C,d
J2	3.488 ± 0.023 B,cd	3.300 ± 0.023 B,c	4.456 ± 0.023 A,b
J3	2.721 ± 0.256 B,ef	2.540 ± 0.110 B,e	3.691 ± 0.110 A,c
J4	1.811 ± 0.153 A,i	1.884 ± 0.126 A,g	0.469 ± 0.126 B,j
J5	0.632 ± 0.074 C,m	1.807 ± 0.104 B,g	2.600 ± 0.104 A,d
J6	2.239 ± 0.221 A,g	2.289 ± 0.171 A,ef	1.757 ± 0.171 B,g
J7	1.983 ± 0.073 A,h	0.970 ± 0.147 B,i	2.434 ± 0.147 A,e
J8	1.135 ± 0.020 A,l	−0.056 ± 0.004 B,m	−0.011 ± 0.104 B,k
J9	−0.144 ± 0.012 A,o	−1.525 ± 0.145 B,n	0.050 ± 0.214 A,k
J10	2.506 ± 0.114 A,f	2.152 ± 0.254 A,f	1.568 ± 0.254 B,h
J11	1.231 ± 0.013 A,k	1.751 ± 0.160 A,h	0.584 ± 0.160 B,i
J12	2.823 ± 0.004 A,e	2.347 ± 0.032 B,ef	2.280 ± 0.032 B,f
J13	0.589 ± 0.217 B,mn	0.015 ± 0.374 B,l	2.755 ± 0.374 A,d
J14	1.457 ± 0.023 A,j	1.172 ± 0.126 A,h	1.553 ± 0.126 A,h
J15	6.910 ± 0.313 A,a	6.870 ± 0.012 A,a	6.360 ± 0.210 A,a
J16	5.068 ± 0.204 A,b	4.839 ± 0.264 B,b	3.796 ± 0.264 C,c
J17	3.414 ± 0.062 A,d	2.991 ± 0.023 B,d	−0.107 ± 0.023 C,l
J18	1.176 ± 0.046 B,l	2.219 ± 0.134 A,ef	2.192 ± 0.134 A,f
J19	−0.584 ± 0.113 C,p	0.784 ± 0.145 B,j	1.517 ± 0.145 A,h
SS	0.418 ± 0.034 A,n	0.530 ± 0.010 A,k	0.580 ± 0.012 A,i

a Capital letters represent significant differences in survival of the same strain under different stress treatments (*P* < 0.05); lowercase letters indicate significant differences in cross-adaptation areas among different strains upon the same treatment (*P* < 0.05).

### Cell membrane integrity of acid-heat-cross-adapted S. aureus.

Membrane integrity is an important factor in maintaining the viability of bacterial cells (23), and it was estimated in this work. As shown in Table 2, the amount of nucleic acid and protein leakage of S. aureus strain J15 with the acid-heat cross-adaptation trait was significantly lower than that (S. aureus strains J19, J9, and J17) without the acid-heat cross-adaptation after being stressed by organic acids (*P* < 0.05). After exposure to acetic acid, lactic acid, and citric acid, the nucleic acid leakage amounts in the control group (S. aureus strains J19, J9, and J17) were 1.29, 1.24, and 1.34 times that of S. aureus strain J15, respectively. After pretreatment with acetic acid, lactic acid, and citric acid, the protein leakage amounts in the control group were 2.25, 1.52, and 2.58 times that of S. aureus strain J15, respectively. It could be that S. aureus strain J15 initiated a coping mechanism upon organic acid stress, including altering the fatty acids in the membrane, enhancing the toughness of the cell membrane, maintaining the cell membrane integrity, and preventing excessive leakage of intracellular substances (24).

**TABLE 2:** Cell membrane integrity of S. aureus after different stress treatments

Leakage	Treatment	Acetic acid			Citric acid			Lactic acid		
		Leakage amt^*a*^		J19/J15 ratio	Leakage amt^*a*^		J9/J15 ratio	Leakage amt^*a*^		J17/J15 ratio
		J19	J15		J9	J15		J17	J15	
DNA (OD_260_)	Acid	0.93 ± 0.12 B,a	0.72 ± 0.26 B,b	1.29	0.87 ± 0.18 B,a	0.70 ± 0.12 B,b	1.24	0.90 ± 0.10 B,a	0.67 ± 0.11 B,b	1.34
	Acid-heat	1.29 ± 0.24 A,a	0.86 ± 0.05 A,b	1.50	1.24 ± 0.07 A,a	0.97 ± 0.04 A,b	1.28	1.22 ± 0.13 A,a	0.74 ± 0.05 A,b	1.65
Protein (OD_280_)	Acid	5.34 ± 0.65 B,a	2.37 ± 0.17 B,b	2.25	4.04 ± 0.60 B,a	2.66 ± 0.33 B,b	1.52	5.76 ± 0.81 B,a	2.23 ± 0.11 B,b	2.58
	Acid-heat	7.43 ± 0.34 A,a	3.01 ± 0.25 A,b	2.47	7.11 ± 0.47 A,a	3.24 ± 0.04 A,b	2.19	6.39 ± 0.31 A,a	3.03 ± 0.15 A,b	2.11

a Different capital letters indicate significant differences of DNA (OD_260nm_)/protein (OD_280nm_) leakage amount of the same strain upon acid and acid-heat treatments. Different lowercase letters indicate significant differences between the acid-heat-cross-adapted strain and control strains upon the same stress treatment.

With further thermal treatment, it was found that after cross-adaptation to acid and heat, the leakage amount of nucleic acids and proteins in the S. aureus strain J15 was still lower than that of the control-group strains (J19, J9, and J17). The nucleic acid leakage amounts in *S. aureus* strains J19, J9, and J17 were 1.50, 1.28, and 1.65 times that of S. aureus strain J15, respectively, and the protein leakage amounts in the control group were 2.47, 2.19, and 2.11 times that of the acid-heat-cross-adapted strain J15, respectively. Mild acidic stress has been shown to increase the expression of heat stress-related genes and to activate heat shock proteins such as DnaK, GroELS, and Clp protease, leading to resistance to heat stress (25).

### The cell membrane potential of cross-adapted S. aureus.

**FIG 1:**
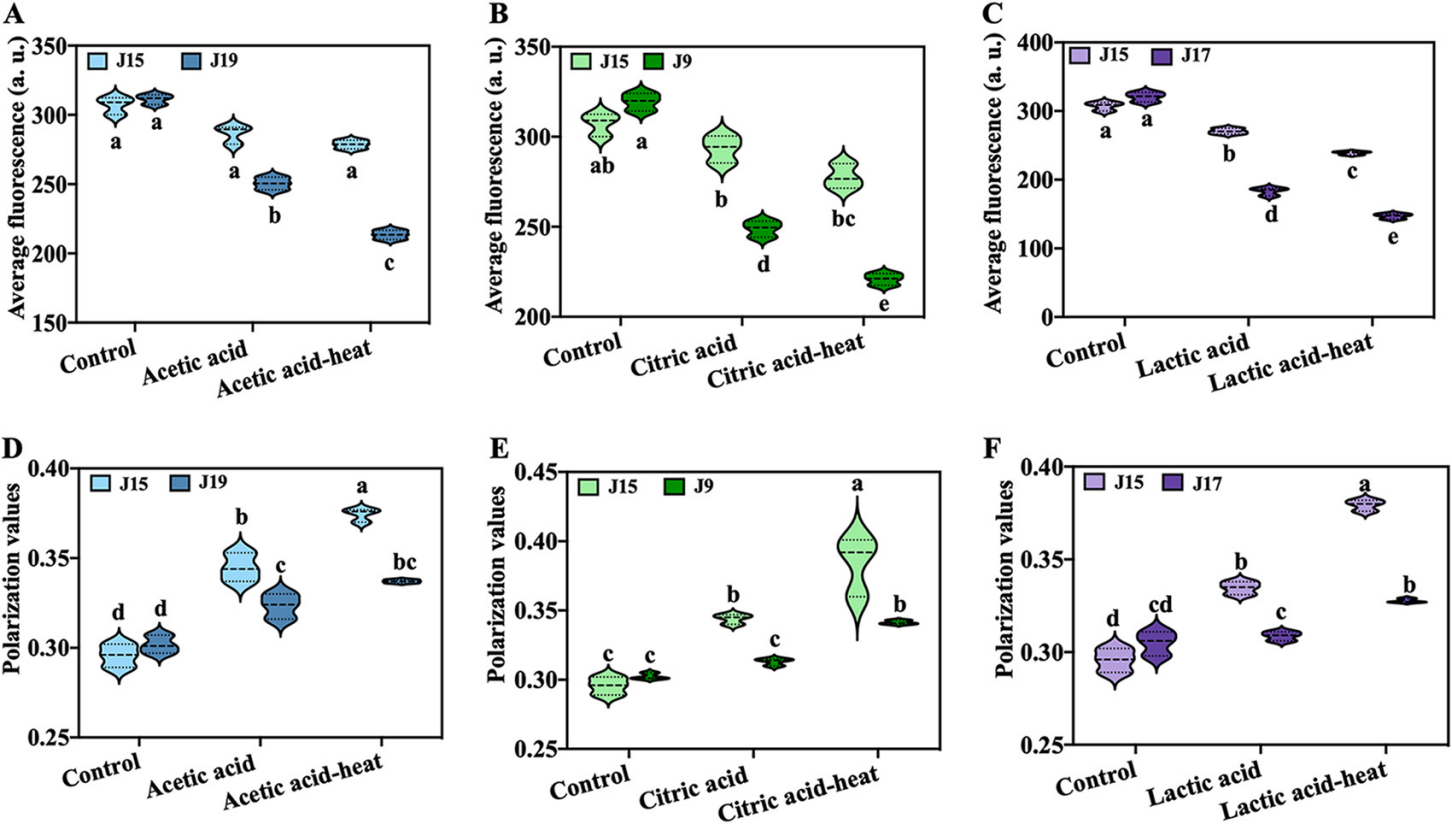
Rhodamine 123 average fluorescence intensities (A to C) (membrane potential) and polarization values (D to F) (membrane fluidity) of Staphylococcus aureus after different treatments. Different lowercase letters indicate significant differences (*P* < 0.05).

Rhodamine 123, a cationic fluorescent dye, was used for monitoring the membrane potential of S. aureus cells. Rhodamine 123 was released when the membrane potential was compromised by external stresses, resulting in a decrease in the fluorescence intensity (26). As shown in Fig. 1A to C, after exposure to acid stress, the membrane potential of all S. aureus strains was decreased compared with that of the unstressed strains. The average fluorescence intensities of rhodamine 123 in S. aureus strain J15 under acetic acid, citric acid, and lactic acid stress were 286.49, 293.41, and 270.62 arbitrary units (AU), respectively, which were significantly higher (*P* < 0.05) than those of the control strains J19, J9, and J17. This result indicated that the variations in the ability to cope with acid stress might result in differences in the membrane potentials of various bacterial strains during a state of cross-adaptation between acid and heat. With subsequent exposure to heat stress, the rhodamine 123 mean fluorescence intensity of S. aureus strain J15 was further decreased, but it was still lower than that of the control strains (J19, J9, and J17) (*P < *0.05) (Fig. 1A to C). Acid-adapted S. aureus strains activated the channels that regulate ion transport on the cell membrane to maintain the charge balance through the transmembrane transport of differently charged ions (27). Under subsequent heat stress, acid-adapted S. aureus strains are able to maintain cell homeostasis, minimize changes in membrane potential, and improve bacterial survival (27).

The cell membrane fluidity serves as a marker for molecular mobility inside the lipid bilayer, and it is strongly correlated with the physiological processes of bacterial cells, including ion transport, growth, and reproduction (28). In this work, the cell membrane fluidity was analyzed by a 1,6-diphenyl-1,3,5-hexatriene (DPH) probe, which could be inserted into the acyl chain of membrane fatty acids. The reduction in cell membrane fluidity could compromise the interaction between cell membrane lipids and DPH molecules. The rotation of DPH was restricted, which led to an increase in the polarization signal of DPH. As shown in Fig. 1D to F, the polarization values of S. aureus strain J15 under acetic acid-, citric acid-, and lactic acid-heat stress were 0.35, 0.34, and 0.34, respectively, while those of the control strains J19, J9, and J17 were 0.32, 0.31, and 0.31, respectively. The polarization value was inversely proportional to the cell membrane fluidity. The cell membrane fluidity of the acid-heat-adapted S. aureus strain was lower than that of the susceptible counterparts. The reduction in membrane fluidity has been attributed to the adaptation to acid stress (29). With subsequent heat treatment, the polarization values of acetic acid-, citric acid-, and lactic acid-stressed S. aureus strain J15 were further enhanced to 0.37, 0.38, and 0.38, respectively, which were higher than those of S. aureus strains J19, J9, and J17. This result indicated that the strains with cross-adaptation phenomenon exhibited decreased membrane fluidity. Alvarez-Ordonez et al. (30) also discovered that acid pretreatment decreased the membrane fluidity (with an increase in the polarization values) of Salmonella strain CECT 4384 by lowering the ratios of unsaturated to saturated fatty acids in cell membrane, which promoted the development of heat tolerance.

### Activities of Na^+^K^+^-ATPase and F_o_F_1_-ATPase of cross-adapted S. aureus.

**FIG 2:**
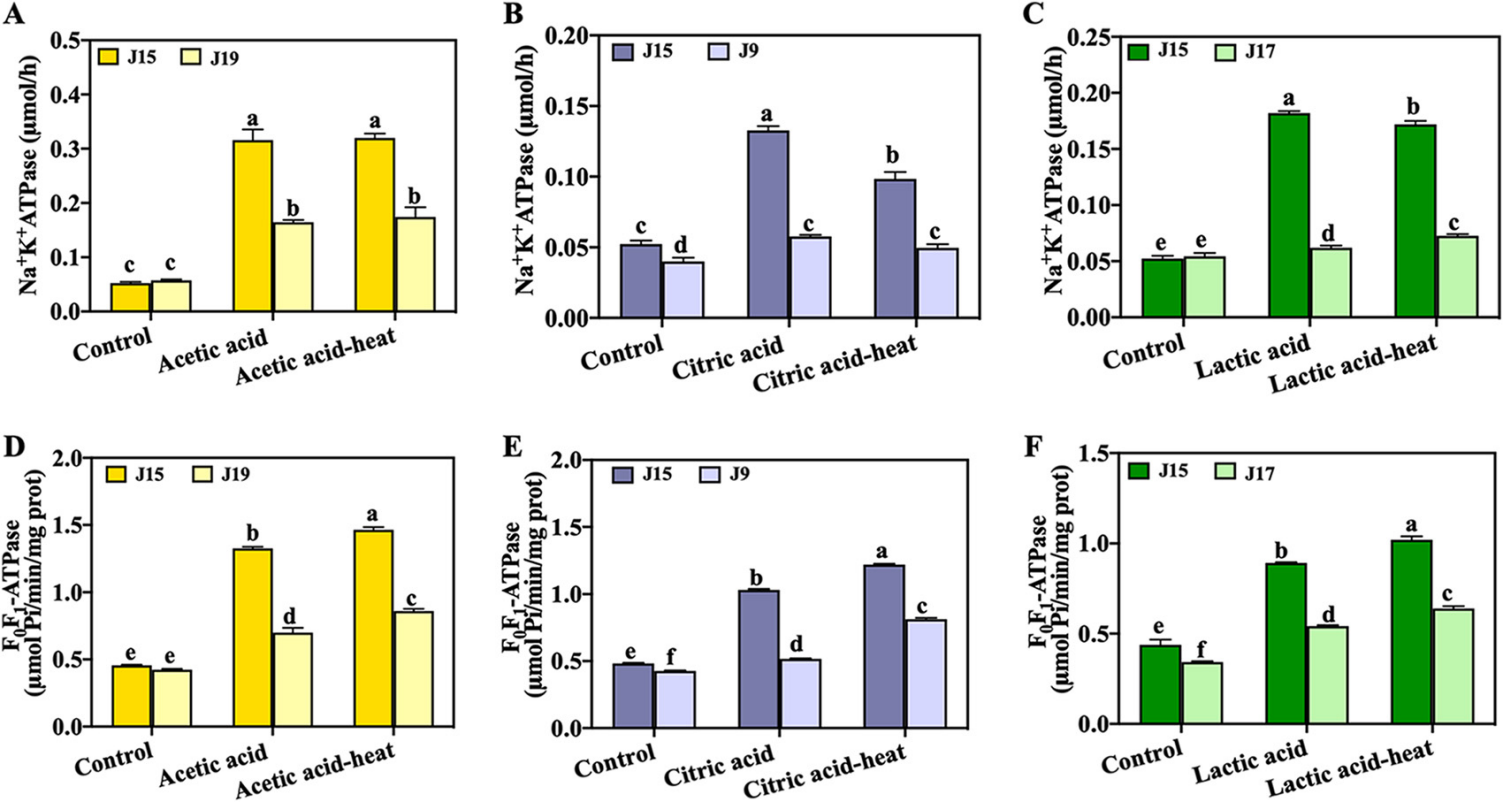
Changes in Na^+^K^+^-ATPase (A to C) and F_o_F_1_-ATPase (D to F) activity in S. aureus cell membrane under different stress treatments. Different lowercase letters indicate significant differences (*P < *0.05).

Na^+^K^+^-ATPase and F_o_F_1_-ATPase are critical transmembrane enzymes that perform critical functions in maintaining the membrane potential, and their activities were evaluated in this work. Na^+^K^+^-ATPase is responsible for exporting sodium ions and importing potassium ions to keep a stable membrane potential in bacterial cells (31). F_o_F_1_-ATPase is a multisubunit enzyme and functions as a membranous channel for proton translocation (32). It plays an important role in the acid adaptation of bacterial cells through the efflux of protons to maintain the internal pH to a normal state (33). As shown in Fig. 2A to C, all strains of S. aureus exhibited an increase in the activity of Na^+^K^+^-ATPase when exposed to acid stress. The enzyme activities of S. aureus strain J15 under acetic acid-, citric acid-, and lactic acid-heat stress were 0.316, 0.133, and 0.182 μmol/h, respectively, while those of the control group (S. aureus strains J19, J9, and J17) were 0.165, 0.058, and 0.062 μmol/h, respectively. The increase in the activity of Na^+^K^+^-ATPase contributed to maintaining the normal function of ion exchange inside and outside the cell, which put S. aureus in a stable physiological state in an acid environment (31). With further thermal treatment, strain J15 under acetic acid-, lactic acid- and citric acid-heat stress had Na^+^K^+^-ATPase activities of 0.320, 0.172, and 0.098 μmol/h, respectively, which were higher than those of strains J19, J9, and J17 (*P* < 0.05).

As shown in Fig. 2D to F, after exposure to acetic acid, citric acid, and lactic acid, the F_o_F_1_-ATPase activity of S. aureus strain J15 was increased to 1.327, 1.031, and 0.892 μmol P_i_/min/mg protein, respectively, which were significantly higher than those of the control strains (*P* < 0.05). Intracellular protons were pumped with the aid of F_o_F_1_-ATPase to maintain the harmony of the interior pH and increase the bacterial survival rate (33). Under acid-heat cross-adaptation, the activity of F_o_F_1_-ATPase in the S. aureus strain J15 was significantly higher than that in the control groups (J19, J9, and J17) (*P* < 0.05). With further heat treatment, as shown in Fig. 2D to F, the F_o_F_1_-ATPase activities of strain J15 with acid-heat cross-adaptation phenomenon were increased to 1.464, 1.221, and 1.021 μmol P_i_/min/mg protein, respectively, which demonstrated that F_o_F_1_-ATPase might play an important role in the development of acid-heat cross-adaptation. F_o_F_1_-ATPase has been reported as the auxiliary chaperone of heat shock protein (HSP) complexes (34). HSP recognizes proteins with abnormal conformations and prevents nonspecific aggregation induced by heat stress (34, 35). Upon acid-heat cross-adaptation, F_o_F_1_-ATPase may be related to the increase in the expression of HSPs, thereby enhancing the resistance of S. aureus to subsequent heat exposure.

### The relative content and change of fatty acid profiles in the S. aureus cell membrane.

Modification of cell membrane fatty acid is one of the important strategies employed by bacterial cells against external stress (36). In this work, the fatty acid profiles of S. aureus before and after treatments were analyzed. The cell membrane of S. aureus consists of straight-chain fatty acids (SCFAs), branched-chain fatty acids (BCFAs), and a small amount of unsaturated fatty acids (USFAs) (Tables 3 to 5). BCFAs were further divided into iso-BCFAs and anteiso-BCFAs (36). The ratio of anteiso-BCFAs to iso-BCFAs was related to the cell membrane fluidity of S. aureus. The methyl groups of anteiso-BCFAs were farther from the end of the fatty acid chain than those of iso-BCFAs. Thus, the decrease in anteiso-BCFAs contributed to the compromise in the cell membrane fluidity (37). When exposed to the organic acids, the ratio of anteiso-BCFAs to iso-BCFAs was significantly decreased in S. aureus strain J15 (*P* < 0.05). The control strains J19, J9, and J17 did not show as great a decrease. The decrease in anteiso-BCFAs was mainly attributed to the reduction of anteiso-C_15:0_ and anteiso-C_19:0_. The increase of iso-BCFAs mainly resulted from the increase of iso-C_15:0_ and iso-C_17:0_. Therefore, the reduction in the ratio of anteiso-BCFAs to iso-BCFAs could explain the decrease in the cell membrane fluidity of strain J15, as indicated above.

**TABLE 3:** Changes of fatty acid composition in the cell membranes of S. aureus upon acetic acid and acetic acid-heat stress

Fatty acid	% change in strain^*a*^					
	J19			J15		
	Untreated	Acetic acid treated	Acetic acid-heat treated	Untreated	Acetic acid treated	Acetic acid-heat treated
13:0 iso	0.26 ± 0.03	0.30 ± 0.02	0.37 ± 0.01	0.38 ± 0.05	0.34 ± 0.03	0.54 ± 0.04
13:0 anteiso	—	0.12 ± 0.01	0.10 ± 0.07	0.18 ± 0.02	0.20 ± 0.01	0.23 ± 0.02
14:00	0.61 ± 0.27	0.82 ± 0.34	0.85 ± 0.31	0.70 ± 0.10	0.55 ± 0.01	0.80 ± 0.04
14:0 iso	1.15 ± 0.06	—	0.85 ± 0.03	1.00 ± 0.26	1.68 ± 0.19	2.06 ± 0.14
15:0 iso	11.05 ± 0.07	11.68 ± 0.14	9.19 ± 0.26	9.96 ± 0.44	12.25 ± 0.25	9.90 ± 0.48
15:0 anteiso	42.42 ± 0.33	43.72 ± 1.98	44.47 ± 1.39	46.07 ± 0.62	43.05 ± 0.55	41.97 ± 0.84
16:00	3.19 ± 1.13	3.31 ± 0.34	3.55 ± 0.67	3.93 ± 0.24	3.05 ± 0.10	3.58 ± 0.33
16:0 iso	1.03 ± 0.05	1.36 ± 0.19	0.64 ± 0.04	0.63 ± 0.03	1.18 ± 0.03	0.94 ± 0.12
17:00	1.18 ± 0.02	1.25 ± 0.23	0.60 ± 0.02	0.54 ± 0.05	1.44 ± 0.07	1.19 ± 0.19
17:0 iso	7.45 ± 0.22	6.55 ± 0.10	6.94 ± 0.16	5.01 ± 0.65	6.44 ± 0.06	5.22 ± 0.59
17:0 anteiso	13.50 ± 0.29	11.90 ± 0.02	11.85 ± 0.24	9.65 ± 0.75	11.67 ± 0.93	10.69 ± 1.13
18:00	6.75 ± 0.03	6.87 ± 0.87	7.13 ± 0.39	8.67 ± 0.38	7.03 ± 0.39	8.75 ± 0.84
18:0 iso	0.51 ± 0.02	0.55 ± 0.04	0.44 ± 0.03	0.35 ± 0.04	0.49 ± 0.02	0.46 ± 0.04
19:00	1.82 ± 0.16	2.04 ± 0.44	1.09 ± 0.14	0.93 ± 0.08	2.17 ± 0.22	2.19 ± 0.23
19:0 iso	2.68 ± 0.05	2.49 ± 0.12	3.46 ± 0.38	3.10 ± 0.20	2.25 ± 0.12	2.42 ± 0.27
19:0 anteiso	3.63 ± 0.24	3.39 ± 0.11	4.21 ± 0.40	4.45 ± 0.15	2.99 ± 0.17	3.91 ± 0.58
20:00	2.76 ± 0.25	3.07 ± 0.45	3.80 ± 0.58	4.18 ± 0.18	2.86 ± 0.26	4.76 ± 0.90
20:0 iso	—	0.15 ± 0.02 a	0.15 ± 0.01	0.18 ± 0.02	0.09 ± 0.07	0.19 ± 0.04
9:00	—	—	—	—	0.04 ± 0.06	—
10:00	—	0.45 ± 0.14 a	0.30 ± 0.06	0.08 ± 0.02	0.23 ± 0.10	0.19 ± 0.02
SCFAs	16.31 ± 1.00 d	17.79 ± 1.97 c	17.33 ± 1.33 c	19.03 ± 0.62 b	17.38 ± 0.97 c	21.47 ± 1.88 a
Anteiso	59.56 ± 0.85 c	59.12 ± 2.01 b	60.63 ± 1.19 a	60.35 ± 0.44 a	57.91 ± 1.29 c	56.80 ± 1.44 d
Iso	24.13 ± 0.16 b	23.08 ± 0.08 b	22.04 ± 0.37 c	20.62 ± 0.25 e	24.71 ± 0.54 a	21.73 ± 1.00 d
Anteiso/iso	2.47 ± 0.02 c	2.57 ± 0.09 c	2.75 ± 0.06 b	2.93 ± 0.01 a	2.34 ± 0.09 c	2.61 ± 0.12 b

a —, not detected. Different letters indicate significant differences (*P* < 0.05) in the ratios of SCFAs, anteiso-BCFAs, or iso-BCFAs or the ratios of anteiso-BCFAs to iso-BCFAs (anteiso/iso).

**TABLE 4:** Changes of fatty acid composition in the cell membranes of S. aureus upon citric acid and citric acid-heat stress

Fatty acid	% change in strain^*a*^					
	J9			J15		
	Untreated	Citric acid treated	Citric acid-heat treated	Untreated	Citric acid treated	Citric acid-heat treated
13:0 iso	—	0.14 ± 0.02	0.21 ± 0.11	0.38 ± 0.05	0.34 ± 0.03	0.50 ± 0.12
13:0 anteiso	—	0.17 ± 0.08	0.11 ± 0.03	0.18 ± 0.02	0.20 ± 0.04	0.17 ± 0.04
14:00	0.18 ± 0.02	0.23 ± 0.04	0.31 ± 0.13	0.70 ± 0.10	0.54 ± 0.02	0.72 ± 0.17
14:0 iso	0.50 ± 0.01	0.73 ± 0.07	0.71 ± 0.10	1.00 ± 0.26	1.84 ± 0.23	2.34 ± 1.05
15:0 iso	9.30 ± 0.25	9.29 ± 0.28	11.05 ± 2.19	9.96 ± 0.44	12.54 ± 0.46	8.98 ± 0.07
15:0 anteiso	44.90 ± 1.05	45.33 ± 1.45	46.61 ± 0.63	46.07 ± 0.62	43.72 ± 1.44	43.20 ± 1.67
16:00	1.22 ± 0.03	1.70 ± 0.20	1.73 ± 0.16	3.93 ± 0.24	2.97 ± 0.07	3.18 ± 0.46
16:0 iso	0.91 ± 0.03	0.91 ± 0.05	0.91 ± 0.04	0.63 ± 0.03	1.26 ± 0.05	1.02 ± 0.19
17:00	0.42 ± 0.02	—	0.62 ± 0.18	0.54 ± 0.05	1.44 ± 0.09	0.85 ± 0.20
17:0 iso	7.83 ± 0.04	7.21 ± 0.10	6.67 ± 0.77	5.01 ± 0.65	6.40 ± 0.11	5.55 ± 1.24
17:0anteiso	20.44 ± 0.08	18.41 ± 0.21	17.30 ± 1.56	9.65 ± 0.75	11.13 ± 0.99	10.50 ± 1.84
18:00	3.60 ± 0.17	4.06 ± 0.28	3.83 ± 0.09	8.67 ± 0.38	6.90 ± 0.54	8.01 ± 1.14
18:0 iso	0.46 ± 0.02	0.54 ± 0.05	0.43 ± 0.07	0.35 ± 0.04	0.50 ± 0.03	0.58 ± 0.05
19:00	0.67 ± 0.08	0.87 ± 0.10	0.61 ± 0.28	0.93 ± 0.08	2.19 ± 0.39	1.83 ± 0.50
19:0 iso	2.65 ± 0.26	3.01 ± 0.36	2.74 ± 0.08	3.10 ± 0.20	2.19 ± 0.27	2.86 ± 0.41
19:0 anteiso	5.18 ± 0.42	5.37 ± 0.51	4.41 ± 0.78	4.45 ± 0.15	2.87 ± 0.30	4.23 ± 0.13
20:00	1.65 ± 0.27	1.77 ± 0.28	1.60 ± 0.10	4.18 ± 0.18	2.85 ± 0.50	4.94 ± 0.97
20:0 iso	—	0.07 ± 0.06 c	0.07 ± 0.09	0.18 ± 0.02	0.12 ± 0.03	0.27 ± 0.08
9:00	—	—	—	—	—	—
10:00	0.09 ± 0.12	0.16 ± 0.17	0.06 ± 0.05	0.08 ± 0.02	—	0.25 ± 0.18
11:00	—	0.02 ± 0.03	—	—	—	—
SCFA	7.83 ± 0.60 c	8.82 ± 0.68 c	8.76 ± 0.33 c	19.03 ± 0.62 a	16.88 ± 1.37 b	19.79 ± 3.61 a
Anteiso	70.52 ± 0.61 a	69.29 ± 0.9 a	68.44 ± 1.87 a	60.35 ± 0.44 b	57.92 ± 1.69 b	58.11 ± 3.49 b
Iso	21.65 ± 0.07 b	21.90 ± 0.23 b	22.80 ± 1.59 b	20.62 ± 0.25 b	25.20 ± 0.66 a	22.11 ± 0.13 b
Anteiso/iso	3.26 ± 0.03 a	3.16 ± 0.07 a	3.00 ± 0.28 b	2.93 ± 0.01 b	2.30 ± 0.12 d	2.63 ± 0.14 c

a —, not detected. Different letters indicate significant differences (*P* < 0.05) in the ratios of SCFAs, anteiso-BCFAs, or iso-BCFAs or the ratios of anteiso-BCFAs to iso-BCFAs (anteiso/iso).

**TABLE 5:** Changes of fatty acid composition of cell membranes of S. aureus upon lactic acid-heat stress

Fatty acid	% change in strain^*a*^					
	J17			J15		
	Untreated	Lactic acid treated	Lactic acid-heat treated	Untreated	Lactic acid treated	Lactic acid-heat treated
13:0 iso	0.30 ± 0.02	0.37 ± 0.14	0.34 ± 0.03	0.38 ± 0.05	0.29 ± 0.02	0.45 ± 0.04
13:0 anteiso	0.22 ± 0.05	0.22 ± 0.06	—	0.18 ± 0.02	0.18 ± 0.01	0.25 ± 0.01
14:00	0.62 ± 0.04	0.66 ± 0.18	0.55 ± 0.07	0.70 ± 0.10	0.45 ± 0.02	0.68 ± 0.07
14:0 iso	1.42 ± 0.04	1.85 ± 0.56	0.88 ± 0.10	1.00 ± 0.26	1.38 ± 0.04	2.32 ± 0.16
15:0 iso	12.23 ± 0.39	10.47 ± 0.46	8.59 ± 0.12	9.96 ± 0.44	12.10 ± 0.16	11.21 ± 1.04
15:0 anteiso	42.46 ± 1.41	42.80 ± 1.29	43.94 ± 0.54	46.07 ± 0.62	44.05 ± 0.68	41.93 ± 0.68
16:00	2.92 ± 0.04	3.62 ± 0.17	2.68 ± 0.12	3.93 ± 0.24	2.82 ± 0.02	3.49 ± 0.28
16:0 iso	1.09 ± 0.03	1.12 ± 0.04	0.75 ± 0.05	0.63 ± 0.03	1.14 ± 0.02	1.19 ± 0.06
17:00	1.37 ± 0.02	1.37 ± 0.07	0.60 ± 0.04	0.54 ± 0.05	1.27 ± 0.03	1.51 ± 0.10
17:0 iso	6.69 ± 0.07	6.10 ± 0.48	7.06 ± 0.11	5.01 ± 0.65	6.45 ± 0.07	6.01 ± 0.07
17:0 anteiso	12.83 ± 0.07	13.10 ± 1.07	13.99 ± 0.15	9.65 ± 0.75	13.20 ± 0.11	11.11 ± 1.20
18:00	7.24 ± 0.46 d	7.27 ± 0.65	6.79 ± 0.17	8.67 ± 0.38	6.50 ± 0.23	7.93 ± 0.12
18:0 iso	0.45 ± 0.02	0.49 ± 0.02	0.48 ± 0.01	0.35 ± 0.04	0.45 ± 0.02	0.52 ± 0.02
19:00	2.09 ± 0.25	1.94 ± 0.53	1.36 ± 0.07	0.93 ± 0.08	1.85 ± 0.09	2.47 ± 0.01
19:0 iso	2.05 ± 0.20	2.14 ± 0.27	2.96 ± 0.17	3.10 ± 0.20	2.02 ± 0.11	2.01 ± 0.11
19:0 anteiso	3.08 ± 0.27	3.31 ± 0.21	4.26 ± 0.49	4.45 ± 0.15	3.13 ± 0.15	2.85 ± 0.39
20:00	2.93 ± 0.45	2.76 ± 0.64	4.36 ± 0.31	4.18 ± 0.18	2.54 ± 0.17	3.54 ± 0.24
20:0 iso	—	0.13 ± 0.02	0.05 ± 0.07	0.18 ± 0.02	0.03 ± 0.04	0.13 ± 0.02
9:00	—	—	—	—	—	—
10:00	—	0.27 ± 0.11	0.35 ± 0.25	0.08 ± 0.02	0.14 ± 0.03	0.37 ± 0.14
11:00	—	—	—	—	—	0.02 ± 0.03
SCFA	17.18 ± 1.18 b	17.89 ± 1.90 b	16.69 ± 0.2 c	19.03 ± 0.62 a	15.58 ± 0.47 c	20.02 ± 0.42 a
Anteiso	58.59 ± 1.03 c	59.44 ± 2.01 c	62.19 ± 0.21 a	60.35 ± 0.44 b	60.56 ± 0.46 b	56.14 ± 0.93 d
Iso	24.24 ± 0.15 a	22.67 ± 0.65 b	21.11 ± 0.01 b	20.62 ± 0.25 c	23.87 ± 0.04 a	23.84 ± 1.21 a
Anteiso/iso	2.42 ± 0.03 b	2.62 ± 0.13 b	2.95 ± 0.01 a	2.93 ± 0.01 a	2.54 ± 0.02 b	2.36 ± 0.15 c

a —, not detected. Different letters indicate significant differences (*P* < 0.05) in the ratios of SCFAs, anteiso-BCFAs, or iso-BCFAs or the ratios of anteiso-BCFAs to iso-BCFAs (anteiso/iso).

In addition, a decrease in SCFAs has also been associated with an increase in membrane fluidity (36). After exposure to acetic acid-, citric acid-, and lactic acid-heat stress, the content of SCFAs in the cell membrane of S. aureus strain J15 increased from 17.38%, 16.88%, and 15.58% to 21.47%, 19.79%, and 20.02%, respectively. The increase in SCFAs was mainly attributed to the increases in C_16:0_ and C_18:0_ in the cell membrane of strain J15. The contents of SCFAs in the control S. aureus strains (J19, J9, and J17) decreased slightly from 17.79%, 8.82%, and 17.89% to 17.33%, 8.76%, and 16.69%, respectively. It has been reported that Gram-positive bacteria such as S. aureus promote the synthesis of SCFAs to reduce the fluidity of cell membranes upon adverse stress (e.g., high temperature) (38). Therefore, the enhancement of SCFAs in cell membranes could contribute to the emergence of the acid-heat cross-adaptation phenomenon.

### The regulation of cell membrane fatty acid synthesis.

In S. aureus, BCFAs accounted for the largest amount of the total fatty acids, and the synthesis of BCFAs performed an important role in the modification of cell membrane fluidity when exposed to external stimuli (39). The synthesis of BCFAs began with the amination of isoleucine, valine, and leucine by the branched-chain-amino-acid aminotransferase BAT (encoded by *ilvE*). The short branched-chain acyl coenzyme A (acyl-CoA) derivatives 2-methylbutyryl-CoA, isobutyryl-CoA, and isovaleryl-CoA were then produced through the oxidative decarboxylation by the branched-chain α-keto acid dehydrogenase BKD (encoded by *lpd*, *bkdA1*, *bkdA2*, and *bkdB*). Subsequently, these acyl-CoA substances were converted into malonyl-CoA, which was further catalyzed to malonyl-ACP by the malonyl-CoA:ACP transacylase FabD (encoded by *fabD*) (40, 41). β-Ketoacyl-ACP was then produced from the β-ketoacyl-acyl-carrier-protein ACP synthase III (encoded by *fabH*)-induced condensation of malonyl-ACP and acetyl-CoA/2-methylbutyryl-CoA to initiate the elongation cycles (involving *fabI*, *fabG*, and *fabF*) to produce BCFAs or SCFAs (42). When subjected to external stress, the bacterial cells developed resistance by regulating the length and saturation of fatty acid chains and changing the composition of fatty acids to alter the membrane fluidity. To further understand the causes of membrane phenotypic changes, the expression of membrane fatty acid synthesis-associated genes in S. aureus under acid-heat stress was investigated in this work.

**FIG 3:**
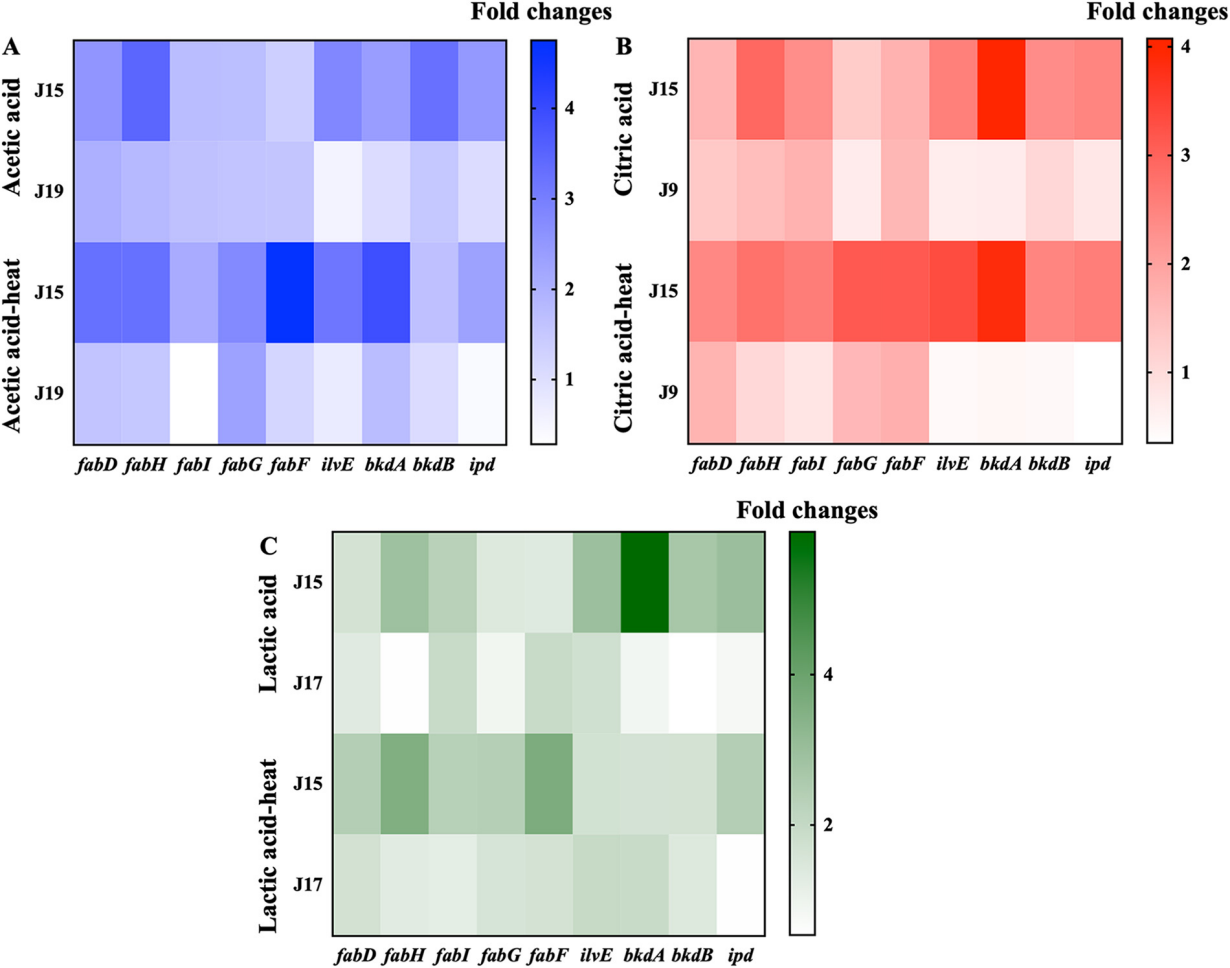
Expression levels of fatty acid biosynthesis genes in the S. aureus cell membrane after various treatments.

As shown in Fig. 3A, after exposure to acetic acid, the *fabD* and *fabH* genes were upregulated by 2.56- and 3.49-fold, respectively, in S. aureus strain J15. The *fabD* and *fabH* genes are responsible for encoding the malonyl-CoA:ACP transacylase FabD and β-ketoacyl-ACP synthase III, which are involved in type 2 fatty acid synthesis pathway for the biosynthesis of BCFAs and SCFAs (42). Genes associated with the biosynthesis of BCFAs were upregulated in strain J15 after exposure to acetic acid. For instance, the gene of *bkdB*, which was shown to be upregulated by 3.26-fold, and is involved in the production of BKD, a multisubunit enzyme complex that consists of a dehydrogenase (E1α), a decarboxylase (E1β), a dihydrolipoamide acyltransferase (E2), and a dihydrolipoamide dehydrogenase (E3) and participates in the early stages of BCFA biosynthesis (43). Further heat treatment dramatically increased the expression of genes (e.g., *fabH*) in strain J15, which might be associated with the enhancement in SCFA content and the changes in the ratio of anteiso-BCFAs to iso-BCFAs measured previously.

After citric acid exposure, the genes involved in the biosynthesis of BCFAs were highly expressed in S. aureus strain J15, which exhibited the citric acid-heat cross-adaptation (Fig. 3B). The *bkdA* gene was significantly upregulated by a factor of 4.08, while the genes related to BCFA synthesis in the control strain did not appear to be changed. The *bkdA* and *bkdB* genes encode the dehydrogenase E1α and the decarboxylase E1β, respectively, which are the polypeptide components of BKD complex and are critical in the synthesis of BCFAs (44). The *fabH* and *fabI* genes in the elongation cycle of the fatty acid biosynthesis were upregulated by 2.91- and 2.34-fold, respectively, in S. aureus strain J15 under citric acid stress. The *fabI* gene encodes an NADPH-dependent trans-2-enoyl-ACP reductase, which reduces 2-enoyl-ACP to fatty acyl-ACP at the expense of NADPH (41). Following heat treatment, as exhibited in Fig. 3B, the genes related to fatty acid biosynthesis were still highly expressed in strain J15.

After lactic acid exposure, the *fabH* and *fabI* genes were upregulated by 2.90- and 2.32-fold, respectively, in S. aureus strain J15. The *ilvE*, *bkdA*, *bkdB*, and *ipd* genes, which are related to the biosynthesis of BCFAs, were also upregulated in strain J15, among which *bkdA* was the most significantly upregulated, 5.89-fold. For the control strain J17, no upregulation was observed in the BCFA biosynthesis-associated genes (Fig. 3C). Acid stress probably activates the fatty acid biosynthesis pathway to adjust the SCFA content or the ratio of anteiso-BCFAs to iso-BCFAs in the cell membrane in order to defend against the acid stress (45). With further thermal exposure, the *fabH* and *fabI* genes were still overexpressed in strain J15, and there was a significant difference (*P* < 0.05) from those of the control strain J17. The expression of the *fabH* gene was upregulated under three organic acid-heat cross-adaptation conditions. It caused changes in the fatty acid profiles of S. aureus, reduced cell membrane fluidity, enhanced heat tolerance, and produced an acid-heat cross-adaptation phenomenon. The *ipd* gene associated with the biosynthesis of BCFAs remained upregulated, while the others were downregulated, as exhibited in Fig. 3C.

### Analysis of heat stress response gene expression.

Heat stress response-associated regulators in bacterial cells include chaperones (DnaK, GroES, and GroEL) under the control of HrcA repressors, the general stress proteins (requiring sigma factors), and the thermoprotease (ClpAP) regulated by the class III stress gene repressor CtsR (35). Pre-exposure to acid stress might induce bacteria to synthesize specific chaperones so as to protect or repair intracellular macromolecules. Heat stress proteins, such as DnaK, DnaJ, GrpE, HrcA, GroEL, GroES, and Clp, have been reported to be crucial acid resistance factors that act as molecular chaperones to promote the repair of nucleic acids and proteins during acid stress, thus maintaining survival through the heat stress (46). The regulation of heat stress response-associated genes in S. aureus under acid-heat stress was explored in this work.

**FIG 4:**
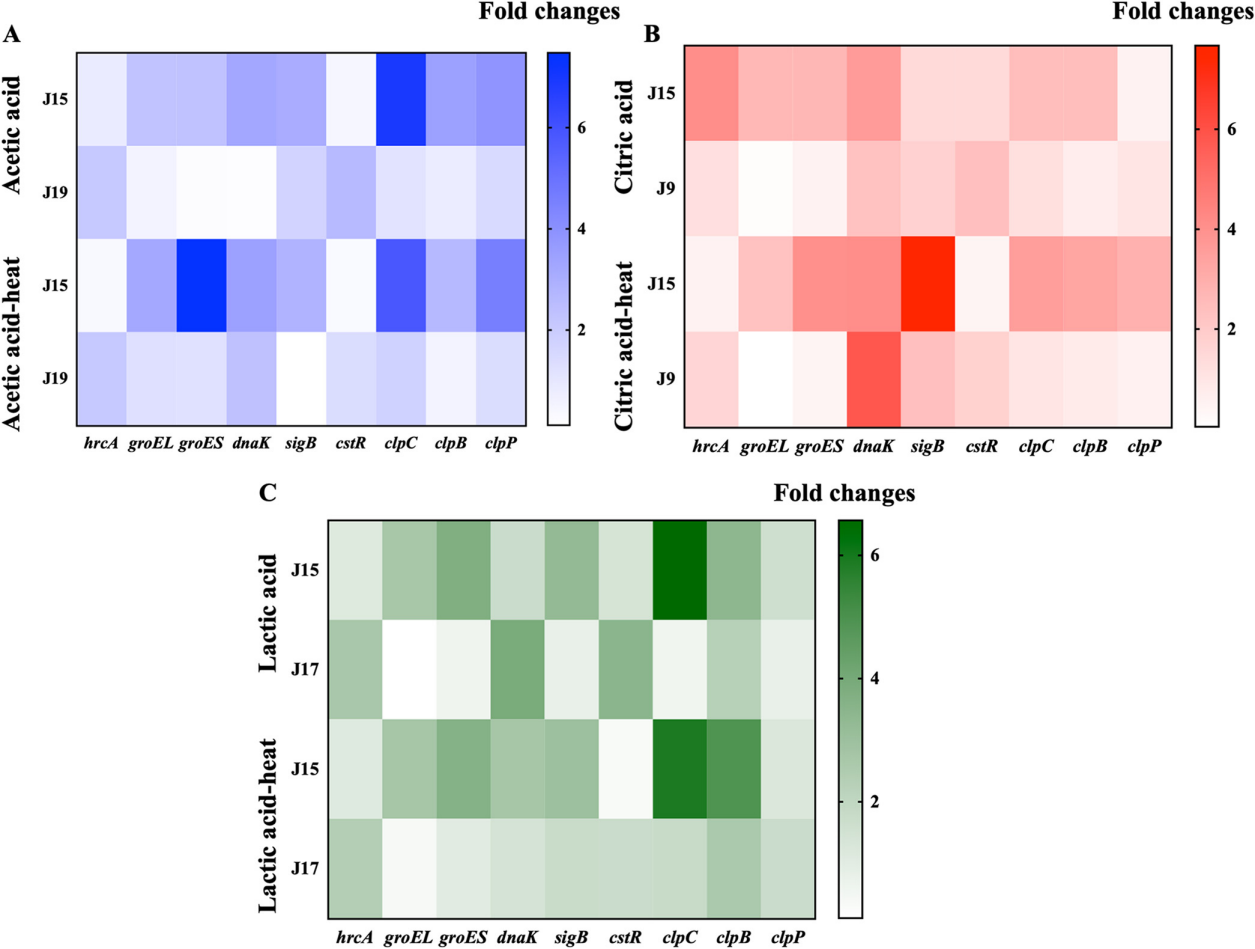
Expression levels of heat shock response genes in the S. aureus cell membrane after various treatments.

As shown in Fig. 4A, the heat stress response-associated genes were upregulated in acetic acid-stressed S. aureus strain J15, except the HrcA and CstR repressor encoding genes (*hrcA* and *cstR*), which were downregulated. The *clpC* gene was the most significantly upregulated, 6.93-fold, in strain J15, after acetic acid stress. DnaK-GroESL operon-associated genes (*dnaK*, *groES*, and *groEL*) were upregulated in strain J15, which was attributed to the downregulation of HrcA and CstR repressors. In S. aureus, both the *dnaK* and *groESL* operons were dually regulated by CtsR and HrcA (47), which could explain the upregulation of *dnaK*, *groES*, and *groEL* and the downregulation of *hrcA* and *cstR* in this work. With further thermal exposure, the heat stress response-related genes remained upregulated in strain J15 (Fig. 4A). The *groES* gene was upregulated by 7.48-fold upon exposure to acetic acid-heat treatment. GroES, an oligomeric protein, contains seven identical subunits, and it is the cochaperonin of GroEL. The GroEL-GroES complex participates in the folding and conformation maintenance of cellular proteins, especially in response to external stress (e.g., high temperature, pH change) (47, 48). The overlapping induction of stress response genes caused by organic acid and heat contributes to the acid-heat cross-adaptation phenomenon in S. aureus.

After exposure to citric acid, most of the heat stress response-related genes were upregulated in S. aureus strain J15 (Fig. 4B). The *groES*, *groEL*, and *dnaK* genes were upregulated by 2.69-, 2.65-, and 3.65-fold, respectively, values which were much higher than those for the control strain J9. With the subsequent heat treatment, the *sigB* and *clpP* genes were upregulated by 7.66- and 2.90-fold, respectively. The σ^B^ factor, encoded by *sigB*, is an important contributor involved in the regulation of a series of genes in response to stimuli (e.g., heat and osmosis) (49, 50). The *clpP* gene encodes the ATP-dependent Clp protease proteolytic subunit, which is involved in the degradation of misfolded proteins induced by an environmental stressor (e.g., high temperature) (51). The expression of *clpP* has been determined to be negatively controlled by CtsR (52). As seen in Fig. 4B, the CtsR-encoding gene *ctsR* was highly repressed in acid-heat-cross-adapted strain J15 cells, which probably contributed to the upregulation of *clpP* gene and prompted acid-heat cross-adaptation in strain J15.

When strain J15 was exposed to lactic acid, the *groEL*, *groES*, and *clpC* genes were upregulated by 2.73-, 3.74-, and 6.56-fold, respectively (Fig. 4C). Similarly, Rode et al. (53) also observed the overexpression of *groEL*, *groES*, and *clpC* in S. aureus upon treatment with lactic acid (pH 4.5) for 180 min. With further thermal exposure, the *groES*, *sigB*, and *dnaK* genes in strain J15 were upregulated by 3.62-, 2.98-, and 2.77-fold, respectively, which might give rise to the heat stress response. The *groES* gene was significantly upregulated in S. aureus strain J15 undergoing the three organic acid pretreatments and remained upregulated upon subsequent heat exposure, suggesting that *groES* may be one of the key contributors to the acid-heat cross-adaptation of S. aureus.

### Conclusions.

**FIG 5:**
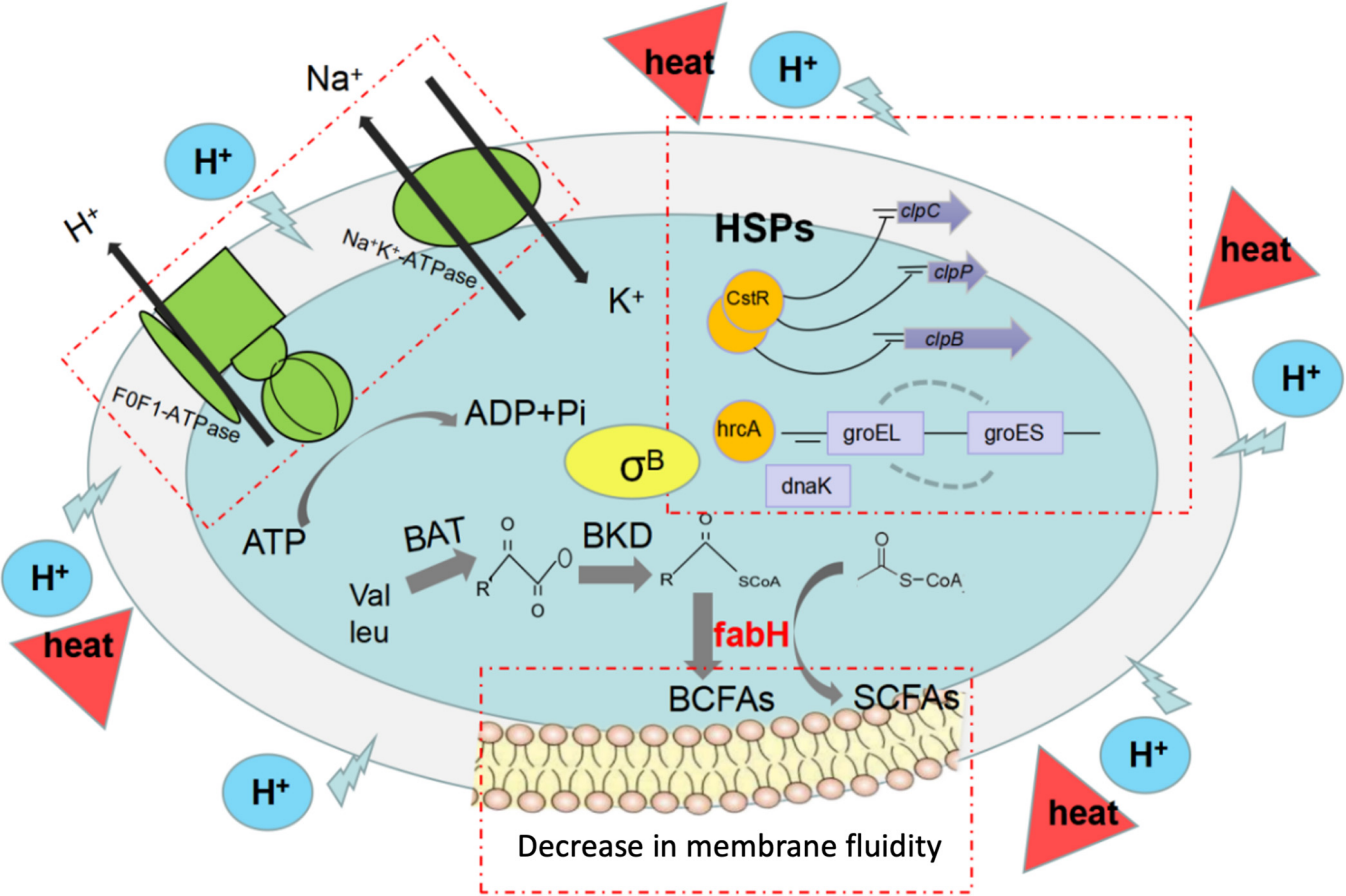
Mechanism of the acid-heat cross-adaptation phenomenon in S. aureus.

In this work, the pretreatment of S. aureus with acetic acid, citric acid, and lactic acid stress induced the development of tolerance to subsequent heat exposure at 60°C. Various S. aureus strains exhibited different acid-heat cross-adaptation areas. Phenotypic traits, such as cell membrane structure and function, enzyme activity, and the fatty acid profile of the cell membrane, were further compared between the acid-heat-cross-adapted strain and acid-heat-susceptible strains (Fig. 5). The acid-induced cross-adaptation to heat might be attributed to the repair of the cell membrane to maintain the membrane integrity and to avoid the leakage of intracellular macromolecules, the equilibrium of the internal and external charge differences to achieve a normal internal pH, and the reduction of cell membrane fluidity through reducing the ratio of anteiso-BCFAs/iso-BCFAs. Gene expression analysis demonstrated that the gene *fabH*, involved in the biosynthesis of fatty acids, as well as *groES* and *groEL*, related to the heat shock response, contributed to the development of the acid-heat cross-adaptation phenomenon in S. aureus strains. The results of this work provide a theoretical basis for optimizing food processing and preventing incomplete sterilization due to the induction of bacterial stress resistance.

## MATERIALS AND METHODS

### Bacterial strains and culture conditions.

S. aureus strain ATCC 25923, designated SS, and S. aureus isolates from food, designated J1 to J19, were used in this study. The sources of the S. aureus isolates are shown in Table 6. All the S. aureus strains were mixed with 50% glycerol solution at a ratio of 1:1 (vol/vol) and stored at −80°C. S. aureus was then recovered on Baird-Parker medium supplemented with 5% egg yolk tellurite emulsion and incubated at 37°C for 24 h. A single colony was transferred to nutrient broth. After incubation at 37°C and 180 rpm for 18 h, S. aureus cells were harvested through centrifugation at 2,320 × *g* for 10 min at 4°C and washed three times by resuspension in sterile phosphate-buffered saline (PBS). The initial concentrations of each S. aureus strain were approximately 10^9^ CFU/mL.

**TABLE 6:** Sources of S. aureus strains used in this work

Strain	Source
SS	ATCC 25923 (American Type Culture Collection)
J1	Yogurt
J2	Chicken
J3	Chicken
J4	Duck
J5	Pork
J6	Mutton
J7	Chicken
J8	Chicken
J9	Chicken
J10	Chicken
J11	Mutton
J12	Chicken
J13	Chicken
J14	Beef
J15	Chicken
J16	Beef
J17	Beef
J18	Chicken
J19	Chicken

### Acid and thermal treatments.

Glacial acetic acid (99.5%), anhydrous citric acid powder, and l-lactic acid (86%) were added to sterile water, and the mixture was adjusted to a pH of 4 with a pH meter (Mettler, Toledo, OH, USA). All the prepared acid solutions were filtered with 0.22-μm syringe filters for microbial removal and stored at 4°C before use. S. aureus was treated with each acid solution, and PBS solution (pH 7.0)-treated S. aureus were used as blank controls. The cells were collected by centrifugation (2,320 × *g*, 25°C, 10 min). S. aureus cells were washed twice by resuspending in sterile PBS and were resuspended in 10 mL of each acid treatment solution. The acid-adapted S. aureus cells were incubated at 60°C for 6 min and then placed in ice to end the thermal stress.

### Microbiological analysis.

After acid treatment, the sample was washed twice by centrifugation at 2,320 × *g* for 10 min at 4°C and resuspended in sterile PBS. The bacterial solution was diluted to an appropriate concentration and plated on tryptone soy agar (TSA) (Hope Bio-Technology Co., Ltd., Qingdao, China), followed by incubation at 37°C for 24 h.

### Ce calculation of the cross-adaptation area.

The inactivation data of S. aureus after exposure to various treatments were fitted to a log-logistic model (54) with the use of Origin 9.1 software (OriginLab, Massachusetts, USA):

$$log\left(\frac{N_{t}}{N_{0}}\right)=\frac{A}{1+e^{4M\left(N-logt\right)/A}}+\frac{A}{1+e^{4M\left(N+6\right)/A}}$$

where *A* represents the maximum inactivation level that can be achieved by acids under certain conditions; *M* is the maximum inactivation rate, log(CFU/mL)/log h; and *N* is the logarithm of the time at which the maximum inactivation rate reachs.

**FIG 6:**
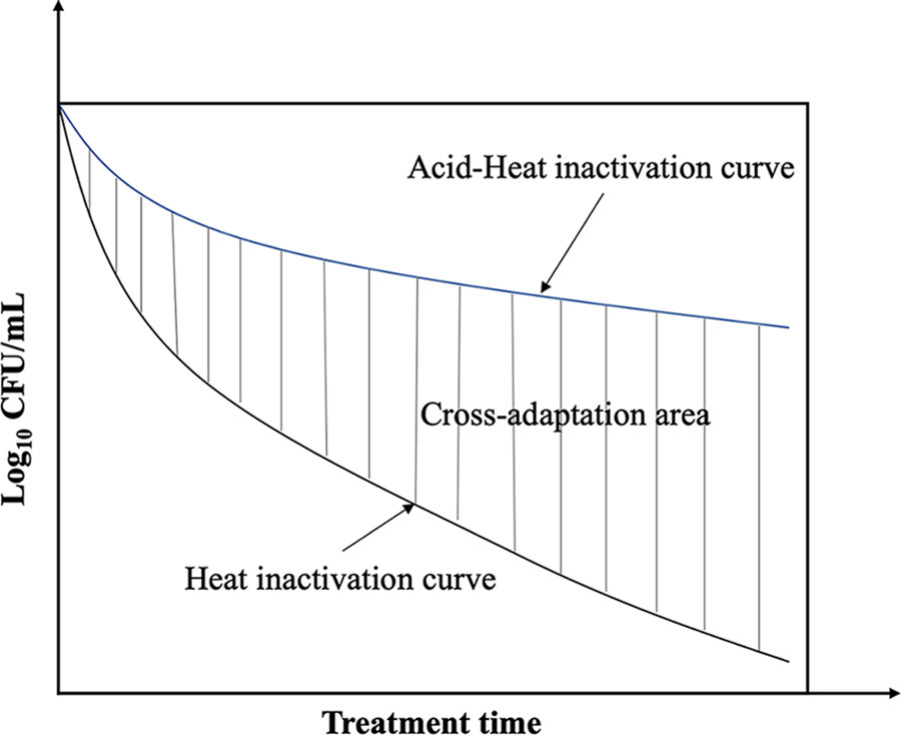
Schematic diagram of the stress cross-adaptation area.

The cross-adaptation area is defined as the area between the stress treatment and control curves, which equals the region under the treatment curve minus the area under the control curve (Fig. 6).

### Analysis of cell membrane characteristics.

**(i) Cell membrane integrity estimation.** After centrifugation at 5,568 × *g* for 2 min, the supernatant was obtained for absorbance measurement at 260 nm and 280 nm with a Nanodrop spectrophotometer (Thermo Fisher, USA), which indicated the nucleic acid and protein leakage amounts for S. aureus, respectively.

**(ii) Cell membrane potential analysis.** Based on the method by Zhang et al. (55) with a minor modification, the untreated and acid-treated groups (0.5 mL) were centrifuged at 2,320 × *g* for 10 min, washed twice with PBS (pH 7.0), and then suspended in 1 mL rhodamine 123 solution (2 μg/mL in PBS) for a 30-min incubation at room temperature. The stained samples were then centrifuged and washed with PBS to remove the excess rhodamine 123. Fluorescence spectra of each sample in the range of 500 to 600 nm were measured with a fluorospectrophotometer (Cary Eclipse, Varian, USA) at an excitation wavelength of 480 nm, and the peak value of fluorescence intensity at an emission wavelength of 530 nm was used to analyze the membrane potential changes (MPC) of bacteria, calculated as *F*/*F*_0_ × 100, where *F* is the fluorescence intensity of the treated sample and *F*_0_ is the fluorescence intensity of the untreated strain.

**(iii) Cell membrane fluidity analysis.** Cell membrane fluidity was measured using the 1,6-diphenyl-1,3,5-hexatriene (DPH) fluorescence probe-based method described by Wang et al. (56) with minor modifications. Bacterial suspensions (approximately 10^8^ CFU/mL) from the treated and untreated groups were centrifuged at 2,320 × *g* for 10 min and resuspended in PBS to adjust the optical density at 600 nm (OD_600_) to 0.5. Then, the DPH probe (2.0 mM) was added, and the mixture was incubated at 37°C in the dark for 1 h. After centrifugation, the supernatant was removed, and the pellets were resuspended in 4 mL of PBS buffer solution. The samples were analyzed by a SpectraMax M5 multifunctional fluorescent plate reader at an excitation wavelength of 360 nm and an emission wavelength of 430 nm (slit width, 5.0/5.0 nm). Fluorescence polarization (*P*) is calculated as (*I_VV_* − *GI_VH_*)/(*I_VV_* + *GI_VH_*), where *G* is the grating factor, *I_VV_* represents the fluorescence intensity obtained when the polarizer and polarizer optical axis in the same vertical direction, and *I_VH_* is the fluorescence intensity obtained when the optical axis of the polarizer and polarizer in vertical and horizontal directions, respectively.

**(iv) Cell membrane fatty acid analysis.** The bacterial suspensions of the treated and untreated groups were centrifuged at 2,320 × *g* for 10 min at 4°C, and the supernatant was discarded. Each sample precipitate was washed twice with PBS buffer. Based on the method of Sasser (57), the saponifying agent (5.13 M sodium chloride in methanol) was added to the bacterial pellets, followed by brief shaking. The mixture was placed into a boiling water bath for 5 min and shaken violently for 5 to 10 s, followed by a 30-min boiling treatment. After cooling to room temperature, 2 mL methylation reagent (3.25 M hydrochloric acid in methanol) was added to the mixture, followed by brief shaking and then incubation at 80°C for 10 min. After cooling, the extract (*n*-hexane–methyl *tert*-butyl ether = 1:1 [vol/vol]) with a volume of 1.25 mL was added and tumbled for 10 min. Subsequently, sodium hydroxide (0.3 M) was added to the remaining organic phase and the upper n-hexane/methyl-tert-butyl ether phase containing the fatty acid methyl esters (FAMEs) was transferred for analysis by Sherlock Microbial Identification System.

### Determination of Na^+^K^+^-ATPase and F_o_F_1_-ATPase activities.

**(i) Na^+^K^+^-ATPase activities.** The bacterial suspensions of the treated and untreated groups were centrifuged at 2,320 × *g* for 10 min, and the precipitate was resuspended in PBS buffer to achieve a concentration of around 10^8^ CFU/mL. Then, the bacterial cells were lysed by ultrasound, and the Na^+^K^+^-ATPase activity was determined with an ultrafine Na^+^K^+^-ATPase kit (Nanjing Jiancheng Institute of Biological Engineering, China). One unit of enzyme activity is the amount of Na^+^K^+^-ATPase decomposing ATP to produce 1 μmol inorganic phosphorus per h per 10,000 bacterial cells.

**(ii) F_o_F_1_-ATPase activities.** F_o_F_1_-ATPase activities were determined using the colorimetric method described by Price and Driessen (28) with minor modifications. The bacterial suspensions of the treated and untreated groups were centrifuged at 2,320 × *g* for 10 min at 4°C, washed with 50 mM piperazine-*N*,*N*′-bis(2-ethanesulfonic acid) (PIPES) buffer, and suspended in 10 mL of PIPES buffer. The bacterial cells were then lysed by ultrasound (300 W) for 25 min. Centrifugation was performed at 4°C, and 2,320 × *g* for 10 min, and the supernatant was obtained for further analysis. The composition of reaction system A included 50 mM KCl, 5 mM MgSO_4_, 10 mM carbonyl cyanide *m*-chlorophenylhydrazone (CCCP), 0.9 mM G-strophanthin, 25 mM HEPES, 1 mM oligomycin, 50 mM KNO_3_, and 4 mM ATP. In the B reaction system, no oligomycin was added, and the other components were the same as those in the A reaction system. The A and B reaction systems were incubated at 37°C for 15 min, and 100 μL of sample was added, followed by a 1-h reaction time. A mixture of 40 mM ammonium molybdate, 1.5 mM malachite green, and 5% (vol/vol) Triton X-100 was added to the A and B reaction systems. After a 1-min reaction, 1.5 mM citric acid was added and incubated for 20 min. A spectrophotometer was used for colorimetry analysis at 660 nm, and the phosphorus content of the solution was calculated according to the phosphorus standard curve. The protein concentrations of the samples were determined with a bicinchoninic acid (BCA) assay kit. F_o_F_1_-ATPase activity (nmol P_i_/min/mg protein) was measured by the amount of inorganic phosphorus released through the hydrolysis by ATPase.

### RT-qPCR analysis of gene expression.

After centrifugation, 1 mL TRIzol reagent was added to the treated and untreated bacterial pellets and oscillated vigorously. Chloroform (0.2 mL) was added and mixed thoroughly, followed by a 5-min incubation at room temperature. After centrifugation at 5,568 × *g* and 4°C for 15 min, the supernatant was transferred to a new tube, and 0.5 mL of isopropanol was added and mixed thoroughly. The pellets were collected after a centrifugation at 5,568 × *g* and 4°C for 10 min, and 75% alcohol was used to wash the pellets three times. After drying, RNase/DNase-free water was added to dissolve the extracted RNA. The integrity and purity of RNA in the sample were determined by gel electrophoresis and absorbance measurements at 260 nm and 280 nm. The extracted RNA was reverse transcribed to cDNA using a SuperScript III first-strand synthesis SuperMix kit (Thermo Fisher, USA). The appropriate diluted cDNA was used for further qPCR analysis. The qPCR system consisted of 25 μL qPCR mix solution (Platinum SYBR green qPCR SuperMix; Thermo Fisher, USA), 1 μL forward/reverse primers, 1 μL cDNA, and 22 μL RNase/DNase-free water. The PCR procedure was as follows: 50°C, 2 min; 95°C, 2 min; 40 cycles of 95°C, 15 s, 60°C, 30 s; and a melting curve analysis. The primers for each gene used in this study were designed with the NCBI Primer BLAST Online system (https://www.ncbi.nlm.nih.gov/tools/primer-blast) and are listed in Table 7. All primers were obtained from Sangon Biotech (Shanghai) Co., Ltd.

**TABLE 7:** Primer sequences of acid and heat response related genes

Function	Gene	Primer direction^*a*^	Primer sequence (5′–3′)
Housekeeping gene	16S rRNA	F	CCAGCAGCCGCGGTAAT
		R	CGCGCTTTACGCCCAATA

Biosynthesis of membrane fatty acids	*fabD*	F	TTG ACG CAT AGT TCG GCA TT
		R	ACT GCA GCC ATG CTT CCT ACA
	*fabF*	F	TTC TGG TAT CGG TGG TAT GGA
		R	CTT GCC CAG TTG CCA TAT CA
	*fabG*	F	GTT GCC GAT GCT GAT GAA GT
		R	TCA TCC CAC TCT TGT TCT TTC A
	*fabH*	F	GAT AAC CGC ACC TGC ACC AT
		R	TGG ATC AAC TTG CAG CAT GTT
	*fabI*	F	GAA GAC TTA CGC GGA CGC TT
		R	TGC TAC CAC CTT CTG GCA TTA
	*ilvE*	F	AGA GAA TGG GTT CCG GAA GG
		R	ACG CCA AGT ACC CCT TCT G
	*ipd*	F	AAT AGC TAC CGT CTG CCC C
		R	AGC TGA AGT CCC TTC CAC G
	*bkdB*	F	ACA CCG CCA AAT GGT
		R	GAA GCT GCG AAA ATG CGT T
	*bkdA*	F	TGC CAG CTG AAA GCA ACT C
		R	CAG CTC ATT CAT CGG ATG ACG

Class I heat shock response	*hrcA*	F	AAT TGT GAT GGC GAA CGC C
		R	TTG GAC AAC CCG TTG GTT C
	*groEL*	F	CCC ACT CGT TTG TAG CAG C
		R	GCT CTG TCA TCG TTG AAC GC
	*groES*	F	TGT TGG AAC ACG ACA CGG
		R	ACG AAG GCG TTA TCG TTG C
	*dnaK*	F	CCA CTA CTT CGT CCG GGT T
		R	AGC AAT GAA AGA CGC TGG C

Class II heat shock response	*sigB*	F	TCGCGAACGAGAAATCATACAATGT
		R	TGCCGTTCTCTGAAGTCGTGA

Class III heat shock response	*cstR*	F	GCGAATATCGCACAGCGT
		R	ACCACCACCACCACGTTT
	*clpC*	F	GAAGAAGCAATTCGTTTAAATCATTCA
		R	CTTTCTAATACTTTTGCAGCAATTCCTT
	*clpB*	F	AGTAGCAGTTAGTGAGCCTGATG
		R	TCTATCTTGAATACGCACACCATG
	*clpP*	F	TGACAACGTAGCAAATTCAATCGTAT
		R	CACTTCCACCTGGTGAATTAATGTAT

a F, forward; R, reverse.

### Statistical analysis.

All experiments were conducted with three replicates. The statistical significance of the obtained data was analyzed through a one-way analysis of variance (ANOVA) and Duncan's multiple-range tests with SPSS 21.0 software (SPSS Inc., IBM Corporation, Armonk, NY, USA). A *P* value of <0.05 was considered statistically significant.
